# Germline *RECQL* gene mutations in Chinese patients with breast cancer

**DOI:** 10.3389/fmed.2024.1366769

**Published:** 2024-02-19

**Authors:** Jun Hu, Yong Shen, Kun Zhang, Yiding Chen

**Affiliations:** ^1^Department of Breast Surgery, The Second Affiliated Hospital, Zhejiang University School of Medicine, Hangzhou, Zhejiang, China; ^2^Department of Breast Surgery, Fuyang Chinese Medicine Hospital, Hangzhou, Zhejiang, China

**Keywords:** breast cancer, susceptibility genes, RECQL, germline mutations, Chinese patients

## Abstract

**Introduction:**

Breast cancer is the most common malignant tumor in women, seriously threatening health and survival. TP-dependent DNA helicase Q1 (RECQL) is a breast cancer susceptibility gene with possible familial links. However, RECQL gene mutations among Chinese women with breast cancer have not been evaluated. Therefore, this study assessed RECQL mutations and their relationships with clinicopathological and epidemiological characteristics in Chinese women with breast cancer.

**Method:**

Clinical information was also obtained via the hospital information system and a follow-up questionnaire. Peripheral venous blood (2 mL) was extracted from all patients and stored at –80°C for future use; the early venous blood samples were from our hospital’s sample bank. RECQL gene sequencing were performed by the Shanghai Aishe Gene Company (China).

**Results:**

We found that a RECQL mutation is a susceptibility factor for breast cancer. Moreover, patients with RECQL mutations were more likely to have a family history of breast cancer than those without. Also, patients with RECQL variants of uncertain significance (VUS) were less likely to develop invasive ductal carcinoma than those without. In addition, unexplained RECQL mutations occurred more often in patients with human epidermal growth factor receptor 2+ breast cancer than in those with other subtypes.

**Discussion:**

These results provide a basis for creating screening criteria specific to Chinese women. However, the frequency of RECQL mutations was low, and the number of pathogenic mutations was too small and could not be analyzed. Thus, more extensive, long-term studies that include other functional experiments are needed to verify these results.

## 1 Introduction

Breast cancer is the most common malignant tumor in women, with a ~ 31% incidence rate (ranking first) and a 15% mortality rate (ranking second) (1). Although the etiology and mechanisms of breast cancer have not been fully defined, many *in vitro* and *in vivo* studies have shown that the occurrence and development of breast cancer are related to various factors, including exogenous and endogenous factors. Exogenous factors include carcinogens, such as physical, chemical, and biological factors of the external environment, personal living, and eating habits. Endogenous factors include immune status, genetic background, and disease history. Genetic factors are some of the most important endogenous pathological factors. Epidemiological survey data show that familial breast cancer cases and hereditary cases account for 15–20% and 5–10% of all cases, respectively (2). Specifically, people with a history of breast cancer among first-degree relatives have a significantly increased risk of developing breast cancer compared to those without a family history. For instance, the 10-year cumulative absolute risk of contralateral breast cancer is 4.3% for those with no breast cancer family history and 8.1% for those with a family history. If a first-degree relative is diagnosed with breast cancer or bilateral breast cancer before the age of 40 years, the risk is three to nine times higher than that of people with no family history (3). In addition, the individual risk of breast cancer is proportional to the number of affected relatives and age at disease onset.

Hereditary breast cancer refers to cancer that carries germline mutations, clinically manifesting as a familial aggregation of breast cancer. Several breast cancer susceptibility genes have been identified, including breast cancer susceptibility genes 1 and 2 (BRCA1 and BRCA2, respectively), partner and localizer of BRCA2 (PALB2), TP-dependent DNA helicase Q1 (RECQL), neurofibromin 1 (i.e., NF1), phosphatase and tensin homolog deleted from chromosome 10 (i.e., PTEN), and tumor protein 53 (i.e., TP53) (4). Breast cancer germline mutations occur in the early stages of embryo development and may originate from parental genetic material; therefore, these mutations are present before birth. BRCA1 and 2 were the earliest discovered breast cancer susceptibility genes and are the genes with the highest penetrance. BRCA1 is on chromosome 17 and contains 24 exons encoding 1,863 different amino acids, whereas BRCA2 is on chromosome 13 and contains 27 exons encoding 3,418 amino acids (5). The mutation frequency in Chinese people is 9.45%, and approximately 20–40% of genetic breast cancer patients have BRCA1/2 germline mutations (6). Moreover, 55–65% of women with BRCA1 mutations and 45% with BRCA2 mutations develop breast cancer before the age of 70 (7). PALB2 has recently been identified as a susceptibility gene with low penetrance. PALB2 interacts with BRCA1 and BRCA2 proteins, contributing to BRCA2 protein stability. Carriers of PALB2 mutations have a four times higher risk of breast cancer than those without (8), and those with PALB2 mutations have a 14 and 35% risk of developing breast cancer at ages 50 and 70 years, respectively (9). Disease-causing mutations that cause loss of PALB2 function have been found in populations in many countries, with detection rates of 0.6–3.9% in individuals with a family history of breast cancer (10).

RECQL, located on chromosome 12, is a RecQ helicase protein family member that encodes DNA helicases and plays an important role in protecting genome integrity (11). Cells lacking RECQL have genomes with higher rates of sister chromatid exchange and are more sensitive to ionizing radiation, resulting in more DNA double-strand breaks (12). The RECQL mutation frequency in familial breast cancer patients is 2.0% versus 0.54% in the common breast cancer population (13, 14). Furthermore, a recent study reported a significantly increased risk of breast cancer among Polish and French-Canadian women with RECQL gene mutations (11).

Early detection, diagnosis, and treatment are the most important means of reducing cancer-related mortality. Identifying high-risk groups for breast cancer, improving tumor prevention awareness, and supervising self-clinical screening rather than extensive population screening are also effective strategies for identifying breast cancer early, reducing social costs, and allocating medical resources appropriately. Ideally, families susceptible to breast cancer, and thus at high risk, would be identified for strict breast cancer monitoring via genetic testing for early identification.

In developed countries, breast cancer susceptibility gene testing and genetic counseling have produced remarkable results regarding early detection. However, screening for breast cancer susceptibility genes in China did not begin until 2012, and a professional hereditary breast cancer evaluation system has not been established. Moreover, clinical data from other countries do not apply to the Chinese population owing to ethnic differences. Therefore, our team used second-generation sequencing to detect whole-exon susceptibility gene mutations and mutations in four breast cancer susceptibility genes (BRCA1, BRCA2, PALB2, and RECQL) in Chinese women. The BRCA1, BRCA2, and PALB2 mutation results have been published separately (15–17). Therefore, this study focuses on the RECQL sequencing results and the clinicopathological and epidemiological characteristics of breast cancer patients with RECQL mutations. Overall, we aim to pro-vide a scientific basis for formulating screening criteria suitable for the Chinese population.

## 2 Materials and methods

### 2.1 Study population

This study included unselected patients with breast cancer admitted to the Second Affiliated Hospital of Zhejiang University School of Medicine between June 2000 and June 2022, regardless of sex, age at onset, or family history. All patients were confirmed to have primary breast cancer based on histopathological findings.

Family history, local recurrence, and distant metastasis data were collected via questionnaires and telephone follow-ups. Peripheral venous blood (2 mL) was extracted from all patients and stored at −80°C for future use; the early venous blood samples were from our hospital’s sample bank.

Clinical information was also obtained via the hospital information system and a follow-up questionnaire, including data on age, age at onset, body mass index, alcohol consumption, smoking status, age at menarche, age at first birth, number of children, number of miscarriages, hypertension, diabetes, oral contraceptives, family history of breast cancer, and history of other malignancies.

**Figure 1:**
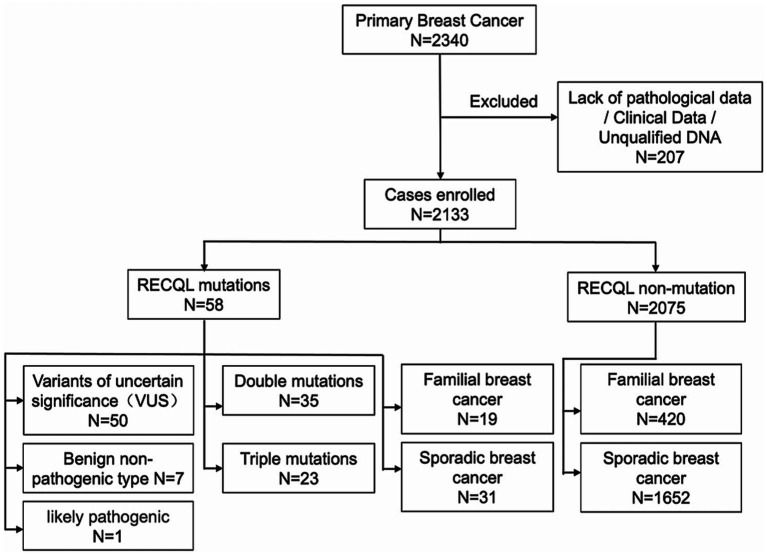
Flow chart of inclusion criteria.

In total, 2,340 blood samples were collected from consecutive patients with primary breast cancer. After the follow-up tests, 206 patients with breast cancer lacking clinical data or pathological records were excluded, and one patient’s sample had poor DNA quality and could not be sequenced. Therefore, 2,133 patients with primary breast cancer were included in this study (Figure 1).

### 2.2 DNA extraction

Genomic DNA was extracted from peripheral blood samples using the QIAamp DNA Blood Mini Kit (Qiagen Inc., Hilden, Germany) or the QIAsymphony DNA Mini Kit (Qiagen Inc.) following the manufacturer’s instructions.

### 2.3 RECQL gene sequencing

Subsequent library quality inspection, computer sequencing, and data analysis were performed by the Shanghai Aishe Gene Company (China). Sequencing was performed using an Illumina HiSeq X10 high-throughput sequencer, and the results were compared with the RECQL reference sequence for mutation detection using the Burrows-Wheeler Alignment tool. Mutation sites were annotated using the Genome Analysis Toolkit (Broad Institute, Cambridge, MA, United States). All pathogenic mutations detected by next-generation sequencing were verified by Sanger sequencing on an ABI3730XL platform (Life Technologies) to rule out false positives.

### 2.4 Statistical analysis

Data were analyzed using GraphPad Prism 9 (GraphPad Software Inc.). To compare the differences between the two groups, we used Mann–Whitney U or two-sided Student’s t-tests as appropriate. We used a Spearman’s rank correlation to analyze the correlation between variables. Results are presented as mean ± SD. Differences of *p < 0.05; **p < 0.01; ***p = 0.001; and ****p < 0.001 were considered statistically significant.

## 3 Results

### 3.1 Baseline characteristics

Of the 2,133 patients enrolled, 298 and 1,835 had familial and sporadic breast cancer, respectively. Patients with familial hereditary breast cancer were required to have a first- or second-degree relative with breast cancer. Of the 298 patients with familial breast cancer, 167 had first-degree relatives with breast cancer, 102 had second-degree relatives, and 50 had both first-and second-degree relatives. Of all patients, 58 had RECQL gene mutations, 50 had a variant of uncertain significance (VUS), seven had benign non-pathogenic breast cancer, and one had a probable pathogenic type. Among them, no patient had only a RECQL gene mutation; however, 35 patients carried double RECQL and BRCA1/BRCA2 mutations, and 23 carried triple RECQL, BRCA1/2, and PALB2 mutations. Of those with triple mutations, one patient carried a BRCA1 mutation, and four carried a BRCA2 mutation.

Moreover, of the 58 patients with RECQL mutations, 19 had a familial genetic background, 31 had sporadic breast cancer, and eight had missing family history in-formation. Of the 2,075 breast cancer patients without RECQL mutations, 420 had a familial genetic background, and 1,652 had sporadic breast cancer. Table 1 presents the clinical characteristics.

**Table 1:** Study population basic information table.

Clinical information	Mean ± SD
age	50.4 ± 9.9
BMI	23.1 ± 3.1
drinker (%)	170 (7.97)
smoker (%)	119 (5.58)
age of menarche	14.5 ± 1.6
age at first birth	26.1 ± 2.9
nullipara (%)	33 (1.55)
age of marriage	24.8 ± 1.5
number of children	1.3 ± 0.8
number of miscarriages	0.8 ± 0.1
oral contraceptive	174 (8.02)
hypertension (%)	218 (10.22)
diabetes (%)	101 (4.74)
family history of breast cancer (%)	301 (14.11)
family history of other cancers (%)	619 (29.02)
*N* = 2,133	

### 3.2 RECQL germline mutation site analysis

The RECQL mutation frequency was 2.719% (58/2133). We identified 16 mutation sites: c.2 T > C, c.1805C > T, c.1063A > G, c.199G > A, c.1088A > G, c.644G > A, c.631A > G, c.1114G > A, c.1361G > A, c.1637 T > C, c.1090G > A, c.1123G > T, c.1211G > C, c.1382A > G, c.700 + 1G > T, and c.1729A > C. The frequency of a benign non-pathogenic germ line mutation was 0.234% (5/2133), and the mutation sites were c.2 T > C, c.1088A > G, and c.700 + 1G > T. Furthermore, 53/2133 patients (2.485%) had RECQL VUS; the corresponding mutation sites were: c.2 T > C (8 times), c.1805 > T; T (6 times), c.1063A > G (1 time), c.199G > A (8 times), c.1088A > G (12 times), c.644G > A (3 times), c.631A > G (1 time), c.1114G > A (4 times), c.1361G > A (3 times), c.1637 T > C (1 time), c.1090G > A (1 time), c.1123G > T (1 time), c.1211G > C (1 time), C.1382A > G (1 time), and c.1729A > C (1 time) (Tables 2, 3).

**Table 2:** BRCA1/2 gene mutation associated with RECQL gene.

Exo	DNA alteration	Protein alteration	BRCA1/2 gene mutation
16	c.2 T > C	p.Met1?	BRCA1(c.4900A > G/c.3548A > G/c.3113A > G/c.2612C > T/ c.2082C > T/c.3448C > T) BRCA2 (c.1114A > C/c.7397 T > C)
16	c.1805C > T	p.Ser602Leu	
16	c.631A > G	p.Arg211Gly	
16	c.1063A > G	p.Thr355Ala	BRCA2 (c.7397 T > C)
16	c.199G > A	p.Ala67Thr	BRCA2 (c.1114A > C/c.7397 T > C)
16	c.1088A > G	p.Asn363Ser	BRCA1 (c2082C > T/c2612C > T/c3113A > G/c3548A > G/ c.4900A > G) BRCA2 (c.865A > C/c.2971A > G/c.7397 T > C/ c.1114A > C)
16	c.644G > A	p.Arg215Gln	BRCA1 (c.3448C > T/c2082C > T/c2612C > T/c3113A > G/ c3548A > G/ c.4900A > G) BRCA2 (c.7397 T > C/c.7522G > A/c.1114A > C/ c.7397 T > C)
16	c.1114G > A	p.Val372Ile	BRCA1 (c2082C > T/c2612C > T/c3113A > G/c3548A > G/ c.4900A > G) BRCA2 (c.7397 T > C/ c.1114A > C)
16	c.1361G > A	p.Arg454His	BRCA1 (/c2082C > T/c2612C > T/c3113A > G/ c3548A > G/ c.4900A > G) BRCA2 (c.7397 T > C/ c.2971A > G/ c.865A > C/c.10234A > G)
16	c.1637 T > C	p.Ile546Thr	BRCA2 (c.7397 T > C/ c.1114A > C)
16	c.1090G > A	p.Glu364Lys	BRCA1 (/c2082C > T/c2612C > T/c3113A > G/ c3548A > G/ c.4900A > G/c.5420 T > C) BRCA2 (c.7397 T > C)
16	c.788C > T	p.Thr263Met	BRCA1 (/c2082C > T/c2612C > T/c3113A > G/c3548A > G/ c.4900A > G/c.2566 T > C) BRCA2 (c.7397 T > C/ c.1114A > C)
16	c.1123G > T	p.Gly375Cys	BRCA2 (c.7397 T > C/ c.1114A > C)
16	c.1211G > C	p.Arg404Pro	BRCA2 (c.7397 T > C/ c.1114A > C/c.10234A > G/c.7691C > T)
15	c.700 + 1G > T		BRCA1 (/c2082C > T/c2612C > T/c3113A > G/c3548A > G/ c.4900A > G) BRCA2 (c.7397 T > C/ c.5785A > G)
16	c.1729A > C	p.Asn577His	BRCA1 (c2082C > T/c2612C > T/c3113A > G/c3548A > G/ c.4900A > G) BRCA2 (c.7397 T > C/ c.5785A > G/c.10234A > G)

**Table 3:** PALB2 gene mutation associated with RECQL gene.

Exo	DNA alteration	Protein alteration	PALB2 gene mutation
16	c.644G > A	p.Arg215Gln	c.1676A > G
15	c.700 + 1G > T	–	
16	c.199G > A	p.Ala67Thr	
16	c.1729A > C	p.Asn577His	
16	c.1361G > A	p.Arg454His	
16	c.1805C > T	p.Ser602Leu	c.1676A > G/c.3114-1G > A
16	c.1361G > A	p.Arg454His	c.925A > G/c.3379 T > C
16	c.2 T > C	p.Met1?	c.3122A > C/c.1676A > G/c.3054G > C
16	c.1114G > A	p.Val372Ile	c.925A > G/ c.1676A > G
16	c.1088A > G	p.Asn363Se	

### 3.3 RECQL mutations and clinicopathological features of breast cancer

The clinicopathological characteristics examined in this study included age at diagnosis, histopathological type, molecular type, tumor size, site of occurrence, lymph node metastasis, estrogen receptor (ER), progesterone receptor (PR), human epidermal growth factor receptor-2 (HER2), Ki-67, the Tumor Node Metastasis (TNM) stage, family history of breast cancer, and family history of other tumors. Table 4 presents the molecular typing criteria.

**Table 4:** Specific criteria for molecular typing of breast cancer.

Molecular subtyping	criterion
Luminal A	ER + and/or PR + , HER2-, Ki67 ≤ 14%
Luminal B	ER + and/or PR + , HER2-, Ki67 > 14%
	ER + and/or PR + , HER2 + , Ki67 at any level
TNBC	ER-, PR-, HER2-
HER2 positive	ER-, PR-, HER2+

#### 3.3.1 RECQL VUSs based on the pathological classification

**Figure 2:**
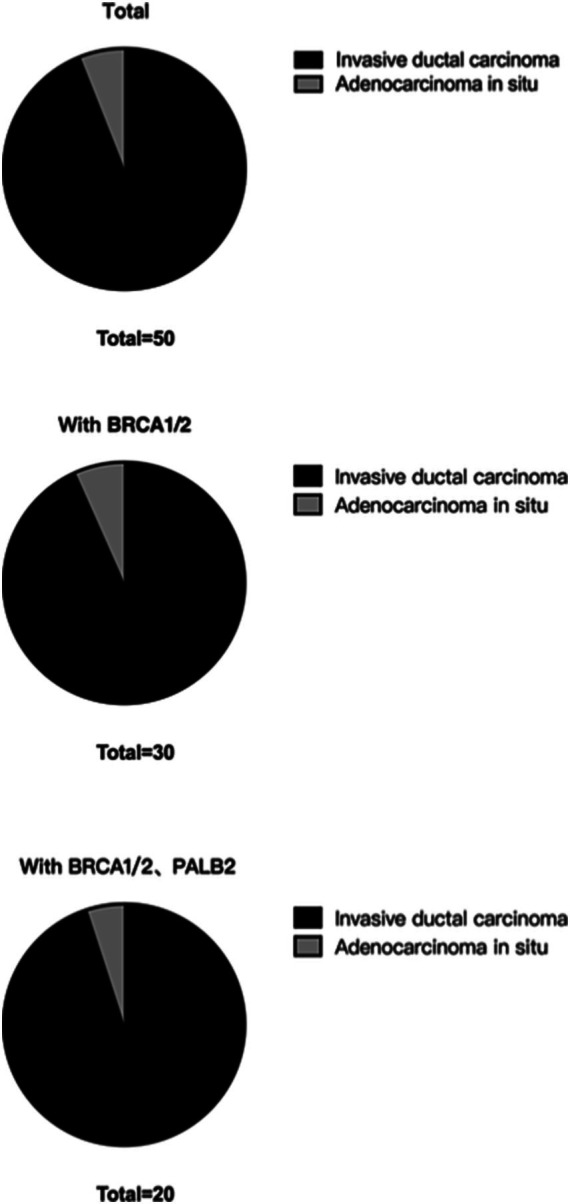
RECQL VUSs in the distribution of subgroups based on pathological classification.

In total, 2,079 of the included patients had pathological classification data. For patients with invasive ductal carcinoma, the BRCA1/2 double mutation frequency was 1.35% (28/2079), and the BRCA1/2 and PALB2 triple mutation frequency was 0.91% (19/2079). For patients with *in situ* carcinoma, the BRCA1/2 double mutation frequency was 0.10% (2/2079), and the BRCA1/2 and PALB2 triple mutation frequency was 0.05% (1/2079; Table 5; Figure 2).

**Table 5:** RECQL VUSs in the distribution of subgroups based on pathological classification.

Group	Number	With BRCA1/2	With BRCA1/2, PALB2
Invasive ductal carcinoma	47	28	19
Carcinoma *in situ*	3	2	1
Total	50	30	20

#### 3.3.2 RECQL VUSs based on the molecular type

**Figure 3:**
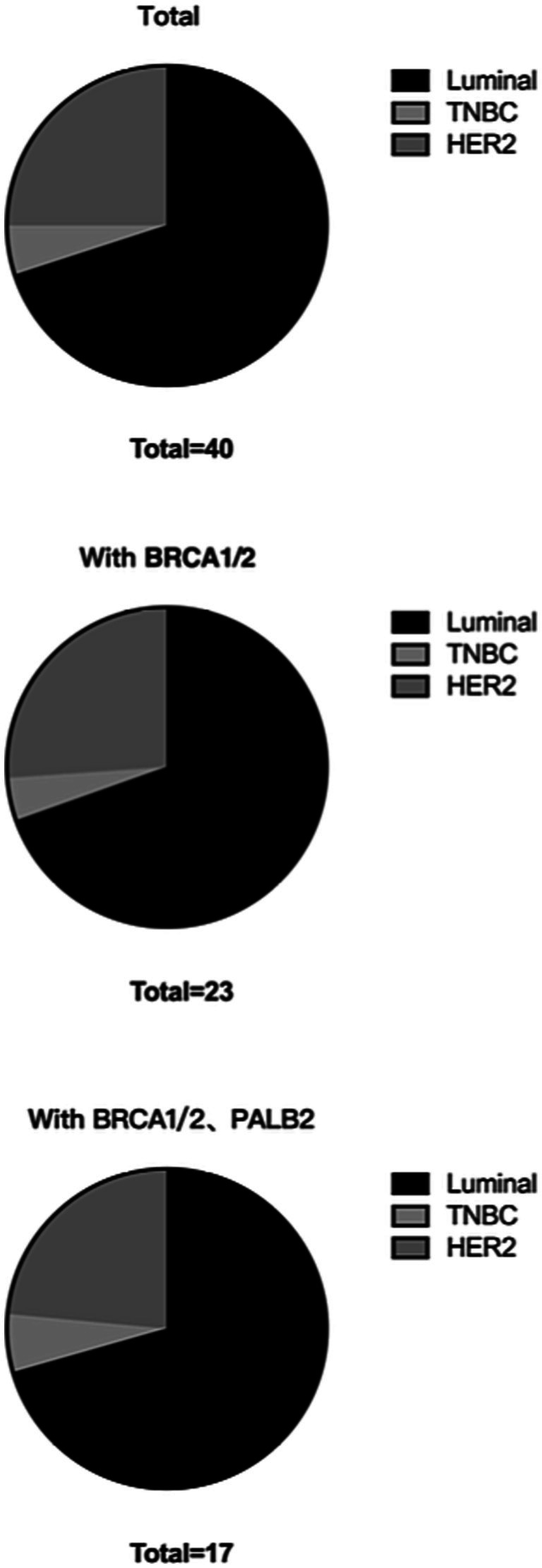
RECQL VUSs in molecular classification of grouping standard distribution of subgroups.

In total, 1,773 patients with complete ER, PR, and HER2 pathology reports were eligible for molecular typing. In the luminal subgroup, the BRCA1/2 double mutation frequency was 0.90% (16/1773), and the BRCA1/2 and PALB2 triple mutation frequency was 0.58% (12/1773). In the triple-negative breast cancer subgroup, the BRCA1/2 double mutation frequency was 0.06% (1/1773), and the BRCA1/2 and PALB2 triple mutation frequency was 0.06% (1/1773). In the HER2+ subgroup, the concomitant BRCA1/2 double mutation frequency was 0.34% (6/1773), and the concomitant BRCA1/2 and PALB2 triple mutation frequency was 0.23% (4/1773; Table 6; Figure 3).

**Table 6:** RECQL VUSs in molecular classification of grouping standard distribution of subgroups.

Group	Number	With BRCA1/2	With BRCA1/2, PALB2
Luminal	28	16	12
TNBC	2	1	1
HER2	10	6	4
Total	40	23	17

#### 3.3.3 RECQL VUSs based on the age of onset

**Figure 4:**
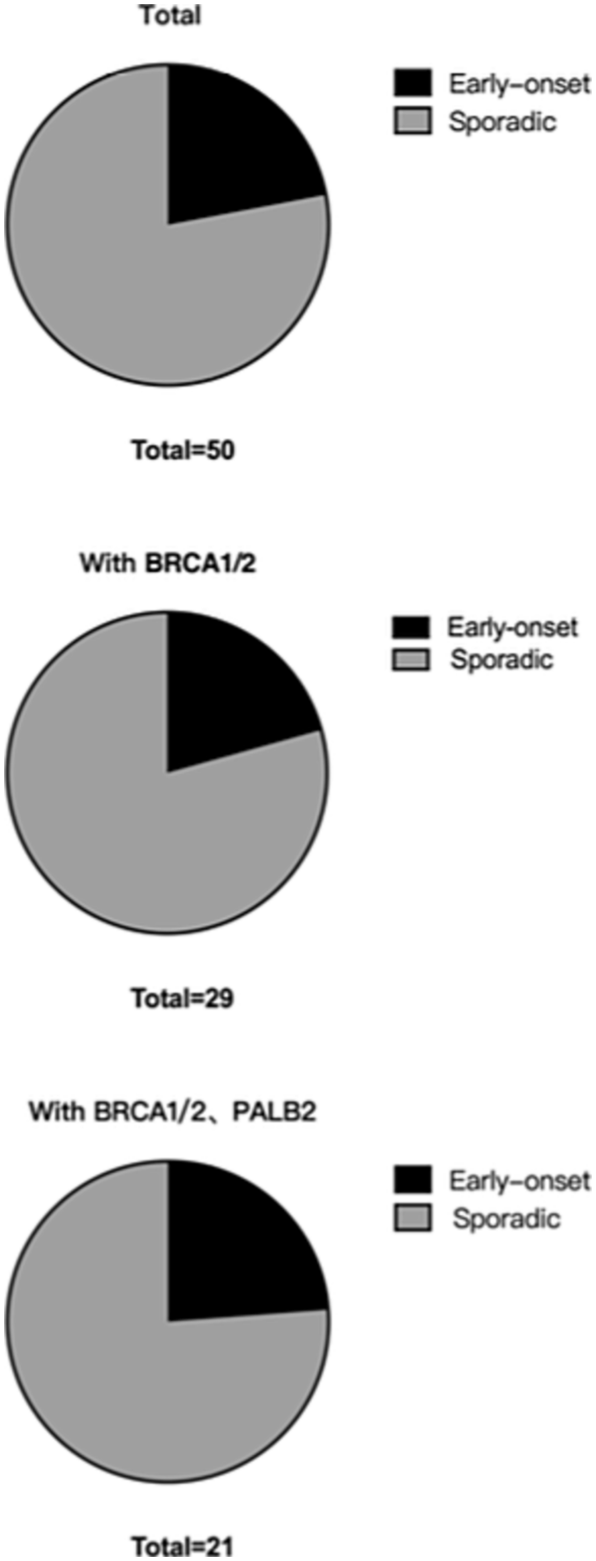
RECQL VUSs in the subgroup distribution based on the age of onset.

For this study, patients aged 40 years or younger at the diagnosis were classified with early-onset breast cancer, and those over 40 years at the diagnosis were classified with sporadic breast cancer. In total, 364 patients had early onset breast cancer; the double mutation frequency was 1.65% (6/364), and the BRCA1/2 and PALB2 triple mutation frequency was 1.37% (5/364). Overall, 1,710 patients had sporadic breast cancer; the BRCA1/2 double mutation frequency was 1.35% (23/1710), and the BRCA1/2 and PALB2 triple mutation frequency was 0.94% (16/1710; Table 7; Figure 4).

**Table 7:** RECQL VUSs in the onset age of the subgroups of grouping standard distribution.

Group	Number	With BRCA1/2	With BRCA1/2, PALB2
Early-onset	11	6	5
Sporadic	39	23	16
Total	50	29	21

#### 3.3.4 Correlations between RECQL gene mutations and clinicopathological characteristics

Patients with RECQL VUS were less likely to develop invasive ductal carcinoma than those without RECQL mutations (91.67% vs. 97.10%). In addition, patients with unexplained RECQL mutations were more likely to occur in patients with HER2+ cancer than those with other groups (*p* = 0.037), suggesting that unexplained RECQL mutations were more common in patients with HER2+ breast cancer. In addition, compared to non-mutated patients, patients with RECQL mutations were more likely to have a family history of other tumors, particularly breast cancer (52.00% vs. 37.31%; 38.00% vs. 20.27%). However, age, histological stage, TNM stage, tumor size, lymph node metastasis, and distant metastasis did not differ between patients with and without RECQL mutations (Table 8).

**Table 8:** Clinicopathological features of RECQL VUSs and non-mutated patients.

	VUS	Nonmutated	*p*
Age			0.374
≤40	11 (22.00%)	386 (18.66%)	
>40	39 (78.00%)	1,683 (81.34%)	
Histopathological types			*<0.001*
Invasive ductal carcinoma	44 (91.67%)	1,676 (97.10%)	
Carcinoma *in situ*	2 (4.17%)	44 (2.55%)	
Other types	2 (4.17%)	6 (0.35%)	
Molecular subtyping			*0.037*
Luminal A	5 (10.42%)	155 (9.04%)	
Luminal B	24 (50.00%)	1,125 (65.64%)	
TNBC	2 (4.17%)	106 (6.18%)	
HER2+	17 (35.42%)	328 (19.14%)	
Histologic staging			0.870
I	9 (19.15%)	334 (18.64%)	
II	27 (57.45%)	960 (53.57%)	
III	9 (19.15%)	434 (24.2%)	
unknown	2 (4.26%)	64 (3.57%)	
TNM			
0	2 (4.00%)	205 (10.07%)	0.083
I	10 (20.00%)	566 (27.80%)	
II	21 (42.00%)	844 (41.45%)	
III	10 (20.00%)	250 (12.28%)	
IV	5 (10.00%)	69 (3.39%)	
unknown	2 (4.00%)	102 (5.01%)	
Tumor size			0.983
≤2 cm	26 (60.47%)	1,030 (60.30%)	
>2 cm	17 (39.53%)	678 (39.70%)	
Lymphonodus			0.520
Positive	36 (78.26%)	1,490 (81.24%)	
Negative	10 (21.74%)	328 (17.88%)	
Unknown	0	16 (0.87%)	
Family history of breast tumors			0.002
Yes	19 (38.00%)	420 (20.27%)	
No	31 (62.00%)	1,652 (79.73%)	
Family history of other tumors			0.034
Yes	26 (52.00%)	773 (37.31%)	
No	24 (48.00%)	1,299 (62.69%)	
Distant metastasis			0.838
Yes	3 (6.00%)	159 (7.64%)	
No	47 (94.00%)	1922 (92.36%)	

Finally, the clinicopathological features did not differ between patients with benign and non-pathogenic variants and those without a mutation, perhaps because of the small sample size (Table 9).

**Table 9:** Clinicopathological features of benign and non-pathogenic variants.

	Benign non-pathogenic	Non-variants	*p*
Age			0.899
≤40	1	386	
>40	5	1,683	
Histopathological types			0.685
Invasive ductal carcinoma	6	1,676	
Carcinoma *in situ*	0	44	
Other types	0	6	
Molecular subtyping			0.821
Luminal A	0	155	
Luminal B	4	1,125	
TNBC	0	106	
HER2+	1	328	
Histologic staging			0.736
I	1	334	
II	4	960	
III	2	434	
Unknown	0	64	
TNM			
0	0	205	0.852
I	1	566	
II	3	844	
III	1	250	
IV	0	69	
Unknown	1	102	
Tumor size			0.750
≤2 cm	4	1,030	
>2 cm	2	678	
Lymphonodus			0.911
Positive	4	1,490	
Negative	1	328	
Unknown	0	16	
Family history of breast tumors			0.682
Yes	1	420	
No	6	1,652	
Unknown			
Family history of other tumors			0.161
Yes	0	773	
No	7	1,299	
Unknown			
Distant metastasis			0.292
Yes	0	159	
No	7	1922	

## 4 Discussion

Recent molecular diagnostic studies have identified RECQL as an important breast cancer susceptibility gene, similar to BRCA1, BRCA2, and PALB2. However, RECQL mutations are infrequent; thus, whether RECQL mutations should be included as a biomarker for pre-onset counseling remains controversial. Large-scale studies on the clinical correlation and pathological characteristics of RECQL mutations are scarce, especially in Asian populations. Therefore, we assessed RECQL mutations and investigated clinical correlations in Chinese patients with primary breast cancer.

In this study, the RECQL mutation frequency was 2.719% (58/2133), occurring much less frequently than BRCA1/2 and PALB2 mutations (18, 19). However, this value is higher than those reported in other countries, including Germany, the United States, and Canada, where the mutation frequency ranges from 0 to 2.6% (11, 20–22). In addition, we did not identify any single mutations in this study; all mutations were accompanied by BRCA1/2 double mutations or BRCA1/2 and PALB2 triple mutations, suggesting that BRCA1/2 or PALB2 may influence RECQL mutations, but this requires further verification. Of the 58 RECQL mutations, 50 were VUSs, seven were benign and non-pathogenic mutations, and only one was a possibly pathogenic mutation. Therefore, more samples carrying definite pathogenic mutations are required for detailed analyses in future studies.

In addition, we identified 16 mutations; the most common mutation frequency was at the c.1088A > G site (12 times), followed by the c.2 T > C and c.199G > A sites (8 times). In a Canadian population, 7 of 1,013 high-risk breast cancer patients and 1 of 7,136 newborns had a c.634C > T mutation (*p* = 0.00004). Moreover, in a Polish population, 30 of 13,136 breast cancer patients and 2 of 4,702 control participants had a c.1667–1,667 + 3delAGTA mutation (*p* = 0.008) (11). These results imply that ethnic differences affect the mutation frequency and mutation sites. Therefore, adequate sequencing and clinical analyses in a Chinese population are urgent for appropriate clinical diagnoses and treatments.

This study is the first to analyze the correlation between RECQL mutations and pathological and clinical features. We found that RECQL VUSs were less likely to cause invasive ductal carcinoma than non-mutated VUSs (91.67% vs. 97.10%). In addition, we found that RECQL VUSs were more common in patients with HER2+ breast cancer than in those with other molecular types (*p* = 0.037). We also found that patients with RECQL mutations were more likely to have a family history of other tumors, particularly breast cancer (52.00% vs. 37.31%; 38.00% vs. 20.27%), than patients without RECQL mutations. However, age, molecular typing, histological stage, the TNM stage, tumor size, lymph node metastasis, and distant metastasis did not differ between patients with and without RECQL mutations. Clinicopathological factors also did not differ between patients with benign and non-pathogenic variants (*n* = 1) and non-variant patients; however, a statistical analysis was not performed owing to the small sample size.

This study has some limitations. First, this study was a single-center study, and few patients had RECQL mutations. Therefore, the sample size should be expanded. Second, the included patients were mainly from the Zhejiang Province; thus, the conclusions may have regional limitations. Finally, based on any form of economic cost–benefit analysis and limitations mentioned above, the results in our study do not yet support screening of whole populations of HER2+ breast cancer patients. These limitations greatly limit the clinical significance of our study.

Nonetheless, compared to other central studies, this study has several advantages. For example, the participants were not subjectively selected (e.g., based on factors such as family history, age of onset, molecular typing, and pathological typing) to avoid selection bias.

## 5 Conclusion

In summary, RECQL mutations are a possible breast cancer risk assessment index, and patients with HER2+ may benefit from RECQL analysis. However, the frequency of RECQL mutations was low, only a few samples with pathogenic mutations were obtained, and this was a single-center study. Therefore, these conclusions are preliminary, and multi-center, large-sample studies including a highly selective patient population are required.

## Data availability statement

The datasets presented in this article are not readily available due to privacy concerns and ethical restrictions. Requests for access to these data should be directed to Kun Zhang, hidrzhangkun@live.com. Access to the data will be provided under conditions that ensure the protection of privacy and confidentiality. Where applicable, interested researchers will be required to sign a data access agreement that specifies the use of data for agreed purpose only.

## Ethics statement

The studies involving humans were approved by Second Affiliated Hospital of Zhejiang University School of Medicine. The studies were conducted in accordance with the local legislation and institutional requirements. The participants provided their written informed consent to participate in this study.

## Author contributions

JH: Conceptualization, Data curation, Investigation, Writing – original draft. YS: Data curation, Investigation, Methodology, Writing – original draft. KZ: Formal analysis, Investigation, Writing – review & editing. YC: Conceptualization, Writing – review & editing.
